# Localisation of Formyl-Peptide Receptor 2 in the Rat Central Nervous System and Its Role in Axonal and Dendritic Outgrowth

**DOI:** 10.1007/s11064-018-2573-0

**Published:** 2018-06-13

**Authors:** Christabel Fung-Yih Ho, Nadia Binte Ismail, Joled Kong-Ze Koh, Saravanan Gunaseelan, Yi-Hua Low, Yee-Kong Ng, John Jia-En Chua, Wei-Yi Ong

**Affiliations:** 1 Department of Anatomy, National University of Singapore, Singapore, 119260 Singapore; 2 Department of Physiology, National University of Singapore, Singapore, 119260 Singapore; 3 Institute of Neurology, University College London, London, UK; 4 Institute of Molecular and Cell Biology, Agency for Science, Technology and Research (A*STAR), Singapore, Singapore 138673; 5 Neurobiology and Ageing Research Programme, National University of Singapore, Singapore, 117456 Singapore

**Keywords:** cPLA2, iPLA2, sPLA2 XIIA, PUFA, Omega-3 fatty acid, Arachidonic acid, Docosahexaenoic acid, DHA, Lipoxin A4, Alox5, Alox15, Resolvin D1, LXR receptor, FPR2, FPRL1, FPR-L1, LXA4R, LXA4 receptor, Pain, Cognition, Brain development, Synaptic plasticity, Learning and memory, Neurite, Neurites

## Abstract

Arachidonic acid and docosahexaenoic acid (DHA) released by the action of phospholipases A_2_ (PLA_2_) on membrane phospholipids may be metabolized by lipoxygenases to the anti-inflammatory mediators lipoxin A4 (LXA4) and resolvin D1 (RvD1), and these can bind to a common receptor, formyl-peptide receptor 2 (FPR2). The contribution of this receptor to axonal or dendritic outgrowth is unknown. The present study was carried out to elucidate the distribution of FPR2 in the rat CNS and its role in outgrowth of neuronal processes. FPR2 mRNA expression was greatest in the brainstem, followed by the spinal cord, thalamus/hypothalamus, cerebral neocortex, hippocampus, cerebellum and striatum. The brainstem and spinal cord also contained high levels of FPR2 protein. The cerebral neocortex was moderately immunolabelled for FPR2, with staining mostly present as puncta in the neuropil. Dentate granule neurons and their axons (mossy fibres) in the hippocampus were very densely labelled. The cerebellar cortex was lightly stained, but the deep cerebellar nuclei, inferior olivary nucleus, vestibular nuclei, spinal trigeminal nucleus and dorsal horn of the spinal cord were densely labelled. Electron microscopy of the prefrontal cortex showed FPR2 immunolabel mostly in immature axon terminals or ‘pre-terminals’, that did not form synapses with dendrites. Treatment of primary hippocampal neurons with the FPR2 inhibitors, PBP10 or WRW4, resulted in reduced lengths of axons and dendrites. The CNS distribution of FPR2 suggests important functions in learning and memory, balance and nociception. This might be due to an effect of FPR2 in mediating arachidonic acid/LXA4 or DHA/RvD1-induced axonal or dendritic outgrowth.

## Introduction

There has been much recent interest in the role of lipid mediators in neuronal signalling. Fatty acids such as arachidonic acid (AA) or docosahexaenoic acid (DHA) are released from membrane phospholipids by cytosolic phospholipase A_2_ (cPLA_2_) and calcium-independent phospholipase A2 (iPLA_2_) [1], and are subsequently metabolized by 15-lipoxygenase (15-LOX-1) and 5-lipoxygenase (5-LOX) to produce lipoxin A4 (LXA4) [2] and resolvin D1 (RvD1) [3] or neuroprotectin D1 [4]. Unlike most other metabolites of arachidonic acid, LXA4 is an anti-inflammatory and pro-resolving lipid mediator that facilitates the resolution of inflammation [5, 6]. It promotes the recruitment of macrophages to clear cellular debris [7, 8], and inhibits macrophage / microglial activation and reduces neuropathic pain [9]. RvD1 is a potent pro-resolvin lipid mediator that limits polymorphonuclear leukocyte recruitment to inflammatory loci [10], and neuroprotectin D1 reduces expression of pro-inflammatory factors and protects human retinal pigment epithelial cells from oxidative stress [11]. Both LXA4 and RvD1 activate a G-protein coupled receptor (GPCR), formyl-peptide receptor 2 (FPR2) (also known as FPR-L1) [12]. The latter is a member of the FPR family of seven transmembrane GPCRs originally identified as anti-microbial receptors on the surface of neutrophils and monocytes/macrophages [13].

FPRs mediate cell chemotaxis in a pertussis toxin-sensitive manner, indicating coupling to the Gi (inhibitory) subfamily of G proteins [13]. The N-formyl group is a crucial determinant of ligand binding to FPR [14]. Since bacterial [15, 16] and mitochondrial proteins [17] are the only sources of N-formyl peptides in nature, it widely thought that these receptors mediate trafficking of phagocytes to sites of bacterial invasion or tissue damage [13]. The FPRs are involved in host defence against bacterial infection, and in the clearance of damaged cells [13]. For example, human FPR2 detects bacterial endotoxins including peptides released by *Staphylococcus aureus*, and specific blocking of FPR2 severely diminished neutrophil detection of endotoxin and leukocyte infiltration [18]. Activation of FPR2 leads to transient calcium ion mobilization [19], ERK phosphorylation [19] and cell motility [20]. Nevertheless, since FPR2 is activated only with high concentrations of N-formyl oligopeptides in the micromolar range in vitro, it is considered a low-affinity receptor for bacterial peptides [21].

FPRs also bind a large and diverse group of lipid and protein ligands with high affinity. For example, FPR2 binds potently to LXA4 in the nanomolar range and is also known as the lipoxin receptor or LXR [12]. Many studies have demonstrated the interaction between FPR2 and LXA4 or RvD1 that mediates the anti-inflammatory, pro-resolving and anti-nociceptive actions of these lipid mediators [10, 22–26]. FPR2 binds to annexin I (lipocortin I) [27], an endogenous glucocorticoid-regulated protein that has anti-inflammatory activities [28, 29]. The presence of three FPR family members, Fpr1, Fpr-rs1 and Fpr-rs2, has been reported in the hippocampus and hypothalamus of the mouse [30, 31]. A survey of the distribution of FPR2 / FPR-L1 has been carried out at the light microscopic level in human organs, tissues and cells, including the brain, and dense staining was reported, but not illustrated, in the cerebellum and brainstem [32]. Treatment of primary murine microglial cells with lipopolysaccharide results in activation of these cells, and this effect is mediated by FPR [13].

There is much recent interest in the role of lipid mediators as signalling molecules in the brain. In particular, recent findings suggest that lipid anti-inflammatory molecules could have a role in processes such as long term potentiation and memory formation [33–37]. However, the contribution of FPR2 to axonal or dendritic outgrowth is unknown. The present study was carried out, to elucidate the expression and localization of FPR2 at the mRNA, protein, immunohistochemical and ultrastructural level in the rat CNS, and to elucidate its role in axonal or dendritic outgrowth, in primary hippocampal neurons.

## Materials and Methods

### Animals

Adult male Wistar rats (250–300 g) were purchased from the Centre for Animal Resources (CARE), National University of Singapore, and housed in temperature controlled (23 ± 1 °C), individually ventilated cages on a 12-h light–dark cycle (7AM-7PM) with access to food and water. Rats were acclimatized for 4 days before the start of experiments. All procedures were in accordance with the Principles of Laboratory Animal Care, and approved by the Institutional Animal Care and Use Committee of the National University of Singapore.

### Real-Time Reverse Transcriptase Polymerase Chain Reaction (RT-PCR)

Four adult male Wistar rats were used for this portion of the study. Rats were deeply anesthetized with a ketamine/xylazine cocktail and sacrificed by decapitation. Ketamine/xylazine mixture used was prepared in saline (ketamine (75 mg/kg), xylazine (10 mg/kg), in 0.9% sodium chloride solution). Various parts of the rat brain including olfactory bulb, prefrontal cortex, striatum, thalamus/hypothalamus, hippocampus, cerebral cortex (labelled CTX1 and CTX2), cerebellum and brainstem were dissected out. The spinal cord was harvested and sub-divided into the cervical [SC(C)], thoracic [SC(T)] and lumbar [SC(L)] segments. CTX1 contained the motor and somatosensory cortices, whereas CTX2 included the parietal association and auditory cortices. Tissues were snap frozen in liquid nitrogen and stored at − 80 °C. Total RNA was extracted using TRizol reagent (Invitrogen, CA, USA) according to manufacturer’s protocol. RNeasy® Mini Kit (Qiagen Inc., Hilden, Germany) was used to purify the RNA. Samples were treated with RNase-Free DNase (Qiagen Inc., Hilden, Germany) to remove any contaminating DNA, and reverse transcribed using High Capacity cDNA Reverse Transcription Kits (Applied Biosystems, CA, USA). Reaction conditions were 25 °C for 10 min, 37 °C for 120 min and 85 °C for 5 s. Real-time PCR amplification was performed using 7500 Real time PCR system (Applied Biosystems) on TaqMan® Universal PCR Master Mix (Applied Biosystems), FPR2 (Rn03037051_gH) and β-actin probes (#4352340E) (Applied Biosystems, CA, USA) according to the manufacturer’s instructions. The PCR conditions were: incubation at 50 °C for 2 min and 95 °C for 10 min followed by 40 cycles of 95 °C for 15 s and 60 °C for 1 min. All reactions were carried out in triplicates. The threshold cycle (CT) was measured as the number of cycles which the reporter fluorescence emission exceeds the pre-set threshold level. Using the ${2^{ - \Delta \Delta {{\text{C}}_{\text{T}}}}}$ method, the relative fold changes were quantified by first obtaining the threshold cycle, CT that inversely correlates with the levels of mRNA present in the sample. All reactions were performed in triplicates, and the mean and standard error calculated.

### Western Blot Analysis

Proteins were first extracted from different regions of the brain and spinal cord using T-PER® Tissue Protein Extraction solution containing 1% HaltTM protease inhibitor and 1% EDTA solution (Thermo Fisher Scientific, IL, USA). Concentrations of the protein obtained were measured using the Bio-Rad protein assay kit. Protein samples (30 µg) were mixed with a loading dye consisting of SDS and DTT, denatured at 95–100 °C for 10 min and resolved in 10% SDS–polyacrylamide (SDS–PAGE) gels under reducing conditions. A protein ladder was used to monitor the electrophoresis run and to check for protein size as well as protein transfer efficacy (Precision Plus Protein Dual Colour Standards, Bio-Rad Laboratories, CA, USA). Resolved proteins were electrotransferred to a polyvinylidene difluoride (PVDF) membrane and non-specific binding sites were blocked by incubation for 1 h with 5% non-fat milk in Tris-buffered saline containing 0.1% Tween-20 (TBST). After blocking, the PVDF membrane was incubated overnight with FPR2 antibody (NLS1878, Novus Biologicals, CO, USA; diluted 1:1000 in 5% non-fat milk in TBST) at 4 °C. Peptide competition assay was carried out with 5 × protein concentration of the peptide antigen (NLS1878PEP, Novus Biologicals, CO, USA). The PVDF membrane was then washed with TBST and incubated with horseradish peroxidase-conjugated anti-rabbit IgG (Thermo Fisher Scientific, IL, USA; diluted 1:2000 in 5% non-fat milk in TBST) for 1 h at room temperature. Protein bands were visualized with an enhanced chemiluminescence kit (Supersignal West Pico, Thermo Fisher Scientific, IL, USA). For loading controls, the membrane was incubated with a stripping buffer for 10 min at room temperature (Restore Western Blot Stripping Buffer, Thermo Fisher Scientific, IL, USA). The membrane was again blocked with 5% non-fat milk in TBST before incubating with a mouse monoclonal antibody to beta-actin (SigmaAldrich, MO, USA; diluted 1:10,000 in 5% non-fat milk in TBST) for 30 min at room temperature. The membrane was then incubated with horseradish peroxidase-conjugated anti-mouse IgG (Thermo Fisher Scientific, IL, USA; diluted 1:10,000 in 5% non-fat milk in TBST) for 30 min. The densities of the 38 kDa band corresponding to the predicted molecular weight of FPR2 without posttranslational modifications, were obtained from the different brain regions, and normalized to that of β-actin, using the Gel-Pro Analyzer3.1 program (Media Cybernetics, MD, USA).

### Immunohistochemistry

Four adult Wistar rats were used for this portion of the study. They were deeply anesthetized and perfused through the left cardiac ventricle with a solution of 4% paraformaldehyde and 0.1% glutaraldehyde in 0.1 M phosphate buffer (pH 7.4). The brains were removed and sectioned coronally at 100 µm using a vibrating microtome (Leica, Wetzlar, Germany). The free-floating sections were washed with repeated changes of phosphate-buffered saline (PBS) for 3 h, and incubated overnight with an affinity-purified rabbit polyclonal antibody to FPR2 (NLS1878, Novus Biologicals, CO, USA), diluted 1:500 in PBS. Peptide competition assay was carried out using antigen-absorbed antibody as described above. Sections were incubated for 1 h in a 1:200 dilution of biotinylated anti-rabbit IgG (Vector, Burlingame, CA), followed by 1 h incubation with avidin–biotin complex. They were stained for FPR2 using a mixture of 3,3′-diaminobenzidine tetrahydrochloride (Sigma-Aldrich, MO, USA) in nickel-Tris buffered saline containing 0.05% hydrogen peroxide. Some of the sections were mounted on glass slides and counterstained with methyl green followed by coverslipping. The remaining sections were processed for electron microscopy.

### Transmission Electron Microscopy

Electron microscopy was carried out by subdissecting some of the immunostained sections of the prefrontal cortex into smaller portions. These were post-fixed with 1% osmium tetroxide, dehydrated in an ascending series of ethanol and acetone, and embedded in Araldite. Thin sections were obtained from the first 5 µm of the sections, mounted on copper grids coated with Formvar, and stained with lead citrate. They were viewed using a JEOL 1010 EX electron microscope (JEOL, Tokyo, Japan).

### Preparation of Rat Hippocampal Neurons

Hippocampal neurons were prepared as previously described with modifications [38]. Postnatal (P)0 or P1 MPF HanTac:WH (Wistar) rat pups were decapitated and hippocampi were rapidly isolated from their brains in a neutral (pH 7.3) dissection solution (mGBSS) containing common salts, glucose and HEPES (Sigma) at 4 °C in 35 mm diameter dishes. Meninges and dentate gyrus were removed prior to the separation of the hippocampus. Following this, dissected hippocampi were enzymatically digested by 0.25% trypsin (Life Technologies, Carlsbad, USA) in a humidified incubator (37 °C, 5% CO_2_) for 30 min. Trypsin was removed and hippocampi slices were triturated up and down for ten times with a siliconized 9 in Pasteur pipette consisting of a fire polished tip opening of approximately 0.7–0.9 mm in diameter. After the hippocampi have settled to the bottom (in about 2 min), the supernatant was collected and the remaining sediments were triturated twice more for 10 and 8 times respectively in 5% serum medium containing MEM Eagle modified (Sigma), glucose, 2 mM glutamine, MEM-vitamin, Mito + Serum extender (Corning, New York, USA) and fetal bovine serum (FBS). The resulting cell suspension was centrifuged at 500 g for 5 min and the cell pellet resuspended in neuronal plating media comprising DMEM/F12 Ham’s Nutrient Mixture (Sigma), 0.5 mM glutamine and B27 (Life Technologies). Cells were plated on HCl-treated glass coverslips coated with poly-D-lysine at a density of 30,000 cells per 12-well plate or 4,000 cells per 96-well plate.

### Cell Viability Assays

Cytotoxicity profiling was performed in 96-well transparent plates with Alamarblue® (Invitrogen®) cell viability reagent. PBP10 and WRW4 were purchased from Tocris Bioscience and diluted in water to obtain 5 and 8 mM stock solutions, respectively. Further dilutions to working concentrations of each compound were achieved by diluting in neuronal plating media. To determine the optimal dosage for each drug, neurons plated in 96-well plates were exposed to a range of concentrations of PBP10 or WRW4 (1.25–10 µM) for 24 h. Non-drug treated samples were treated with vehicle control (water). PBS was used to wash each well twice prior to the addition of 100 µl of Alamarblue® mixture consisting of 10 µl Alamarblue® reagent and 90 µl of neuronal plating media. After 4 h of incubation in the dark, fluorescence from individual wells was measured using the TECANTM Reader (Infinite® 200 PRO series) employing excitation and emission wavelengths of 570 and 585 nm, respectively.

### Axon and Dendrite Growth Assays

Hippocampal neurons grown 1 day in vitro (DIV) were treated with 5 µM of PBP10 or 7.5 µM of WRW4 for 3 days. Control samples were treated with vehicle control (water). Cells were fixed with 3.7% paraformaldehyde (Sigma) at the end of 3 days and permeabilised with 0.3% Triton-X (Sigma). The fixed samples were then rinsed with PBS and blocked with 10% normal goat serum (Merck) at room temperature for 30 to 45 min. Cells were incubated with primary antibodies against MAP2 (polyclonal rabbit anti-MAP2, Synaptic Systems, dilution 1:400) and Tau (polyclonal guinea pig anti-Tau, Synaptic Systems, dilution 1:400) at room temperature for 1 h before three consecutive washes with PBS to remove excess antibodies. Following this, cells were incubated with secondary antibodies (Cy2-conjugated donkey anti-rabbit, dilution 1:200; Cy3-conjugated donkey anti-guinea pig, dilution 1:400; both from Jackson ImmunoResearch) for 1 h at room temperature. Cells were further washed with PBS prior to mounting onto glass slides with Fluoro-Gel II with DAPI (Electron Microscopy Sciences).

### Quantification of Axon and Dendrite Lengths

Images of individual neurons per treatment (that is, with or without drugs) were acquired and tiled using a Zeiss Axio Observer Z1 Microscope equipped with a motorised stage. Total axon and dendrite lengths were measured using the semiautomatic tracing function in the NeuronJ plugin of ImageJ. Possible differences in neurite lengths between drug-treated (PBP10 or WRW4) and vehicle treated controls, were analysed using one-way ANOVA with Dunnett’s post-test using GraphPad Prism 6.0 software. p < 0.05 was considered significant.

## Results

### Expression of FPR2 mRNA in the Rat CNS

**Fig. 1:**
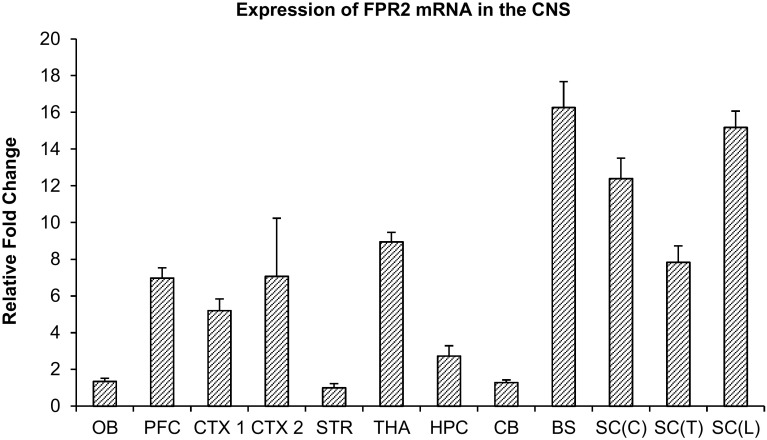
Real-time RT-PCR analysis of FPR2 mRNA expression in various parts of the rat brain including olfactory bulb (OB), prefrontal cortex (PFC), primary somatosensory cortex (CTX1), parietal association cortex and secondary auditory cortex (CTX2), striatum (STR), thalamus and hypothalamus (THA), hippocampus (HPC), cerebellum (CB), brainstem (BS), cervical spinal cord [SC(C)], thoracic spinal cord [SC(T)], and lumbar spinal cord [SC(L)].Fold change values were normalized to the lowest expressing FPR2 mRNA in the striatum. Data represents mean and standard error from 4 Wistar rats

The relative expression levels of FPR2 mRNA in the brain and spinal cord were determined by real-time RT-PCR (Fig. 1). Results showed that the striatum had the lowest level of FPR2 mRNA expression. Hence all fold changes were normalized to that of the striatum. The brainstem (BS) had the greatest FPR2 mRNA expression level, followed by the spinal cord lumbar [SC(L)], spinal cord cervical [SC(C)], thalamus/hypothalamus (THA), spinal cord thoracic [SC(T)] and prefrontal cortex (PFC). Low levels of FPR2 mRNA were found in the olfactory bulb, hippocampus, cerebellum and striatum (Fig. 1).

### Expression of FPR2 Protein in the Rat CNS

**Fig. 2:**
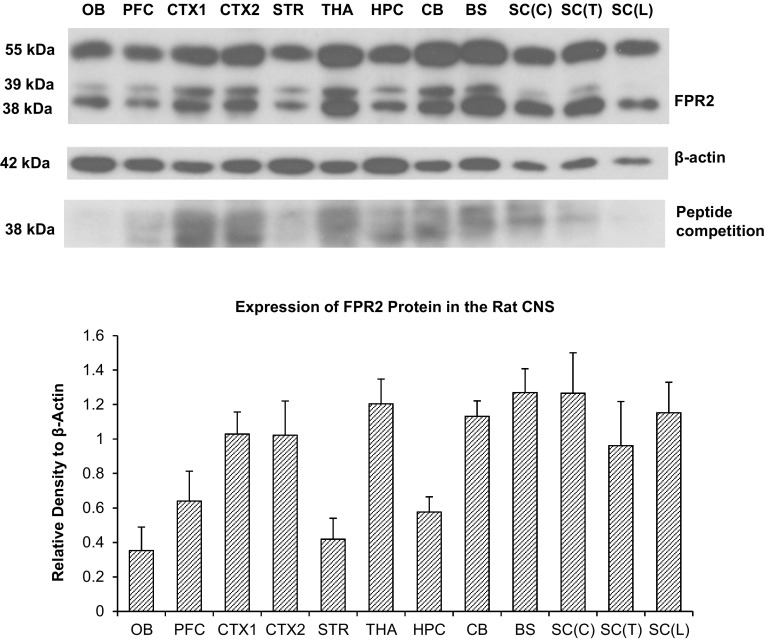
**a** Immunoblot of FPR2 protein in various parts of the rat brain including olfactory bulb (OB), prefrontal cortex (PFC), primary somatosensory cortex (CTX1), parietal association cortex and secondary auditory cortex (CTX2), striatum (STR), thalamus and hypothalamus (THA), hippocampus (HPC), cerebellum (CB), brainstem (BS), cervical spinal cord [SC(C)], thoracic spinal cord [SC(T)], and lumbar spinal cord [SC(L)]. Blots incubated with antigen-absorbed antibody i.e. peptide competition, show reduced band intensities. **b** Densities of the 38 kDa band corresponding to the molecular weight of FPR2 without posttranslational modifications normalized to that of β-actin. Data represents mean and standard error from four Wistar rats

Western blot analysis showed that the FPR2 antibody recognised two major bands and one diffuse band. Blots incubated with antigen-absorbed antibody showed reduced density of the bands, indicating specificity of the antibody (Fig. 2a). The major band of approximately 38 kDa and the diffuse band of 39 kDa represent the non-glycosylated form of FPR2. The other major 55 kDa band is likely a glycosylated form of FPR2. Quantitative densitometric analysis of the 38 kDa band corresponding to the molecular weight of FPR2 without posttranslational modifications, revealed the brainstem had the highest expression, followed by the cervical spinal cord, thalamus/hypothalamus, lumbar spinal cord, cerebellum, primary and secondary motor cortex and the primary somatosensory cortex (CTX1), parietal association cortex and secondary auditory cortex (CTX2), thoracic spinal cord, prefrontal cortex, hippocampus, striatum and olfactory bulb (Fig. 2b).

### Immunohistochemistry

**Fig. 3:**
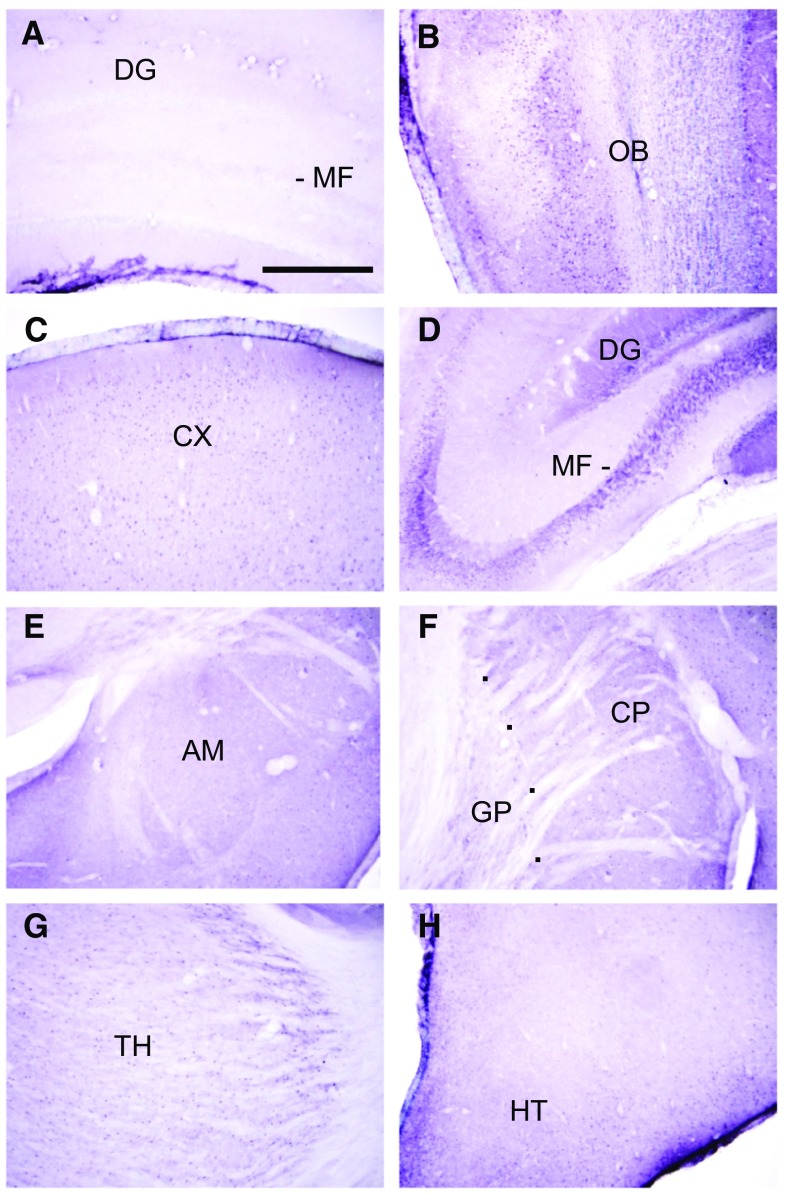
Sections from the forebrain. **a** Sections labelled with antigen-absorbed antibody, showed absence of labelling. **b** Moderately dense staining is present in the olfactory bulb. **c** The cerebral cortex (CX) is moderately labelled. Staining is present in punctuate profiles in the neuropil. **d** The cell bodies, dendrites (DG) and axons (mossy fibres, MF) of dentate granule neurons are densely labelled. **e** The amygdala (AM) is lightly labelled. **f** The caudate-putamen (CP) is lightly labelled. The globus pallidus (GP) is very lightly labelled or unlabelled. **g** The thalamus is lightly labelled. **h** The hypothalamus (HT) is moderately densely labelled. Scale = 0.5 mm

**Fig. 4:**
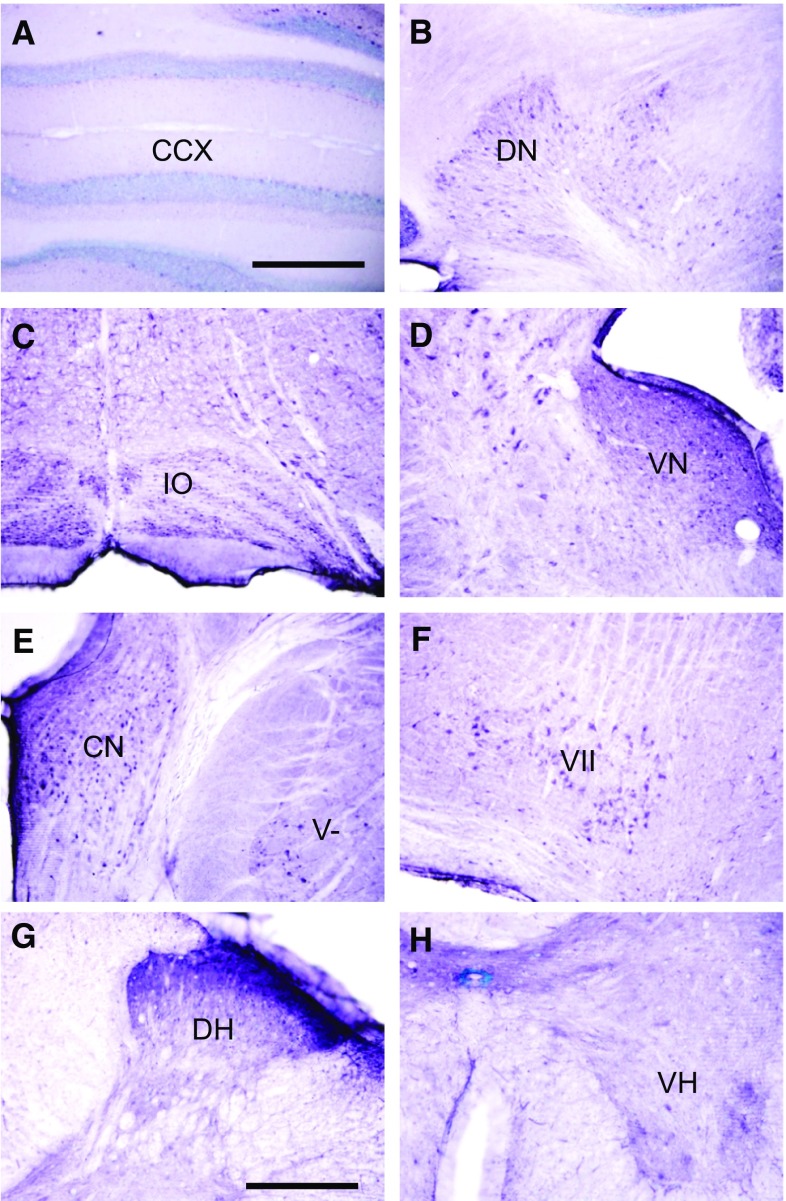
Sections in the hindbrain and spinal cord. **a** The cerebellar cortex contained scattered, lightly labelled Purkinje neurons. **b** Dense staining is present in the deep cerebellar nuclei, including the dentate nucleus (DN). **c** The inferior olivary nucleus (IO) is densely labelled. **d** The vestibular nuclei (VN) are densely labelled. **e** The dorsal and ventral cochlear nuclei (CN), and the spinal trigeminal nucleus (V) are densely labelled. **f** The facial motor nucleus (VII) is densely labelled. **g** The dorsal horn of the spinal cord (DH) is densely labelled. **h** The ventral horn (VH) of the spinal cord is moderately densely labelled. Scale: **a**–**f**: 0.5 mm. **g, h** = 250 µm

Sections incubated with antibody neutralised by the blocking peptide showed absence of immunostaining, indicating specificity of immunoreaction (Fig. 3a). Moderately dense staining was observed in the olfactory bulb (Fig. 3b). The cerebral cortex was moderately labelled (Fig. 3c). Little or no staining was observed in neuronal cell bodies. Instead, staining was observed as punctuate profiles in the neuropil. These were found to be mostly axon pre-terminals at electron microscopy (see below). The hippocampal formation was lightly stained, except for the cell bodies, dendrites, and axons of dentate granule neurons (mossy fibres) which were very densely labelled. The mossy fibres appeared as a characteristic band in the stratum lucidum of CA3 (Fig. 3d). The amygdala (Fig. 3e) and striatum (Fig. 3f), including the caudate putamen (Fig. 3f) were lightly labelled. As with the cerebral neocortex, label was mostly absent from cell bodies, but present as punctuate profiles in the neuropil. The globus pallidus was very lightly labelled or unlabelled (Fig. 3f). The thalamus was lightly labelled (Fig. 3g), while the hypothalamus was moderately labelled (Fig. 3h). The cerebellar cortex contained scattered labelled Purkinje neurons, but was otherwise unlabelled (Fig. 4a). Dense staining was, however, observed in the deep cerebellar nuclei (Fig. 4b), and nuclei in the brainstem that are connected to the cerebellum, including the inferior olivary nucleus (Fig. 4c) and vestibular nuclei (Fig. 4d). The dorsal and ventral cochlear nuclei (Fig. 4e), the spinal trigeminal nucleus (Fig. 4e) and the facial motor nucleus (Fig. 4f) were densely labelled. Dense labelling was observed in the superficial portion of the dorsal horn (Fig. 4g), while moderate staining was found in the ventral horn (Fig. 4h) of the spinal cord.

### Transmission Electron Microscopy

**Fig. 5:**
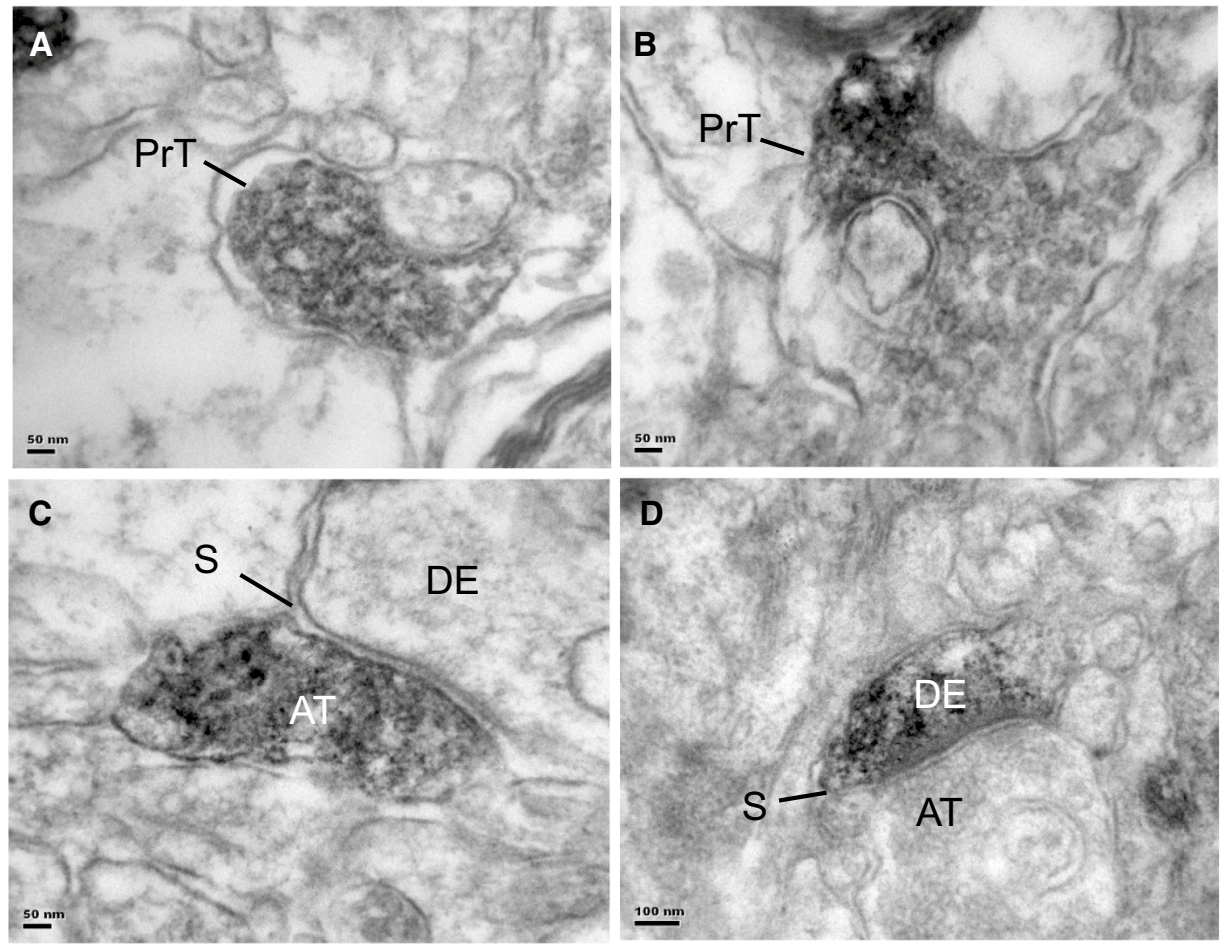
Electron micrographs of FPR2 immunostained sections from the prefrontal cortex. **a, b** Immunostaining is mostly present in axon pre-terminals (PrT) that did not form synapses with postsynaptic structures. **c** Occasional axon terminals (AT) are observed to form asymmetrical, putatively glutamatergic synapses (S) with unlabelled dendrites (DE). **d** Occasional labelled dendrites (DE) are also found, that formed asymmetrical synapses (S) with unlabelled axon terminals (AT). Scale: **a, b, c** = 50 nm, **e** = 100 nm

Electron microscopy of immunostained sections of the prefrontal cortex showed FPR2 label was mostly present in axon pre-terminals with small round synaptic vesicles that did not form synapses with dendrites (Fig. 5a, b). Occasional labelled axon terminals were observed to form asymmetrical, putatively glutamatergic synapses with unlabelled dendrites (Fig. 5c), and occasional, labelled dendrites were found that formed asymmetrical synapses with unlabelled axon terminals (Fig. 5d). Glial cells and blood vessels were unlabelled.

### Inhibition of FPR2 Signalling Reduces Axon and Dendrite Growth

**Fig. 6:**
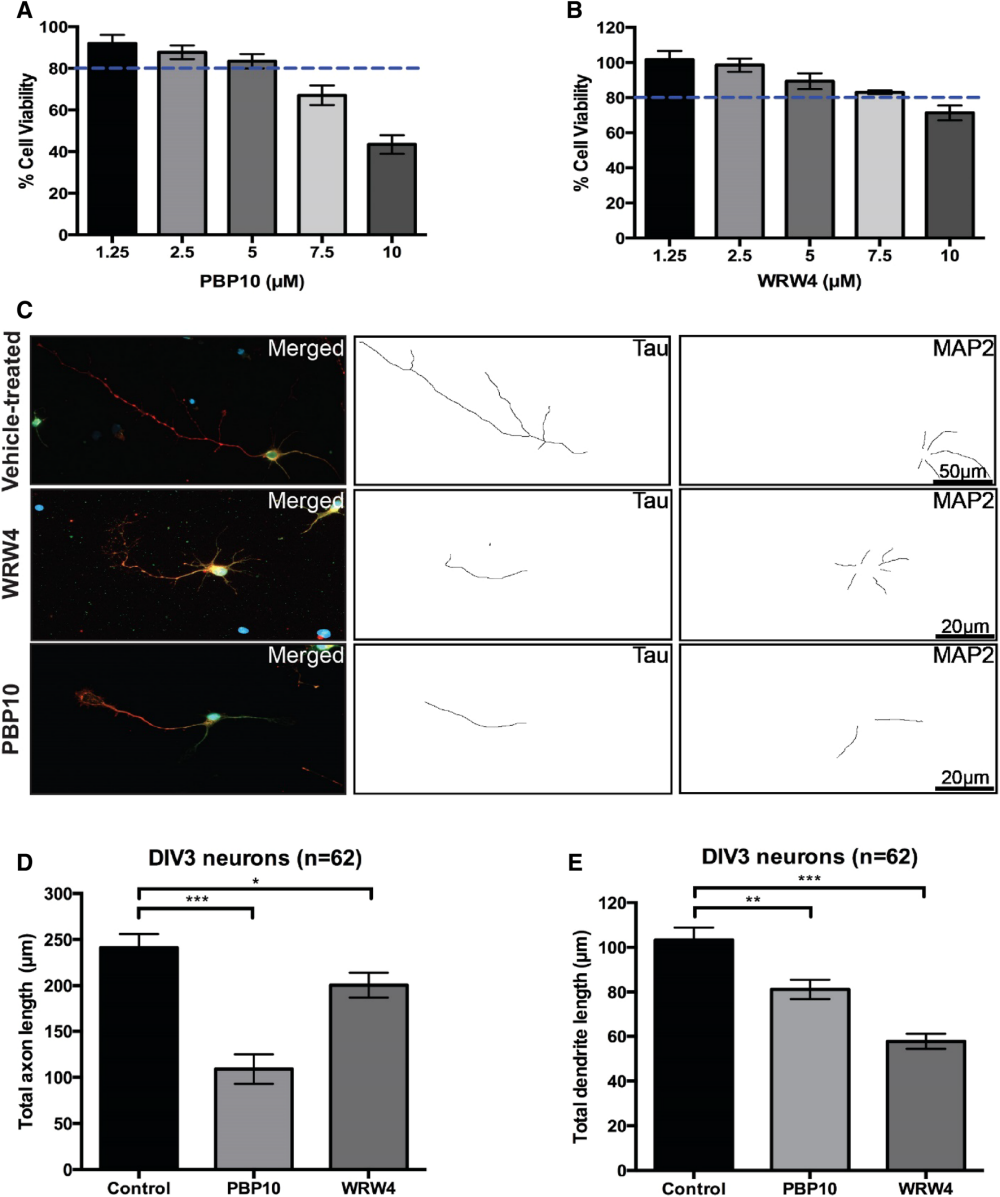
Treatment with WRW4 and PBP10 negatively affects axon and dendrite outgrowth in developing hippocampal neurons. Hippocampal neurons were exposed to increasing doses of PBP10 (**a**) or WRW4 (**b**) a day after plating and cell viability was evaluated 1 day after treatment. Drug concentrations below 5 and 7.5 µM showed more than 80% cell viability (indicated by the blue dotted lines) for PBP10 and WRW4 respectively. **c** Representative immunofluorescence microscopy images showing Tau (an axonal marker, in red) and MAP2 (a dendritic marker, in green) in drug-treated (PBP10 or WRW4) or vehicle-treated neurons with their respective axon and dendrite traces. Quantitative analyses of mean total dendrite length (**d**) and mean total axon length (**e**) in drug- or vehicle-treated neurons. While both WRW4 and PBP10 reduced axonal and total dendritic lengths in comparison to the vehicle-treated control, PBP10 demonstrated a stronger effect in reducing the axon length while WRW4 exhibited a larger influence in decreasing total dendritic length. Data was derived from two independent experiments. Error bars represent SEM. Scale bar in **c** correspond to 50 µm. Statistical analyses in panels **d** and **e** were performed with one-way ANOVA and corrected using Dunnett’s post-test. ***p < 0.001; **p < 0.01; and *p < 0.05

To examine the possible roles of FPR2 in neurons, we exposed developing hippocampal neurons to the FPR2 inhibitors WRW4 or PBP10. WRW4 is a six amino acid peptide that has been shown to block FPR2 signalling by inhibiting the binding of FPR2 agonists [39]. PBP10 is a ten-amino acid long, membrane permeable peptide derived from gelsolin that inhibits FPR2 signalling by blockade of several FPR2-mediated downstream processes [40, 41]. We postulated that inhibition of FPR2 signalling could affect axonal outgrowth in developing neurons. To determine this, we exposed primary hippocampal neurons to increasing doses of WRW4 or PBP10 to determine the optimal concentration to be used for each drug. We decided to expose the neurons to 5 µM (PBP10) and 7.5 µM (WRW4) since more than 80% of the treated neurons remained viable when analysed by the Alamarblue cell viability assay, when the drugs were used at or below these concentrations (Fig. 6a, b). Strikingly, we observed that total axonal length was significantly decreased in drug-treated neurons (Fig. 6c, d). Interestingly, PBP10 appeared to exert a much greater effect than WRW4 (54.8 ± 4.7% vs. 17.2 ± 3.6% reduction, respectively). This result is consistent with previous findings that PBP10 negatively impacts actin assembly [40, 42]. Remarkably, neurons exposed to both drugs also demonstrated significant reductions in total dendritic length (Fig. 6e). Importantly, the Alamarblue cell viability assay detects the metabolic activity of a cell based on the oxidation–reduction (REDOX) potentials of molecules involved in the electron transport chain, which takes place in the mitochondria [43]. Given that both mitochondrial function and homeostasis are intact with the dosage used, local demands of ATP supply and calcium buffering capacity are therefore normal. This indicates that the viable cells are still able to grow normally with no effects from cell impairments contributing to the reductions in total axonal and dendritic lengths. Thus the reductions in total axonal and dendritic length are directly due to the effects of inhibiting FPR2 signalling when exposed to WRW4 or PBP10. Results are consistent with recent studies which show that FPR2 is expressed in hippocampal neural stem cells, and promotes differentiation into neurons with more and longer primary neurites per cell [44, 45].

## Discussion

The present study was carried out to elucidate the expression and distribution of FPR2 in different regions of the brain and spinal cord. Real-time RT-PCR results indicate that FPR2 mRNA is generally present in all brain regions, with higher expression in the brainstem and spinal cord than the forebrain. In particular, expression in the brainstem is double that of the cerebral cortex, and 16 times that of the striatum. The results of Western blots are generally consistent with that of RT-PCR, and high protein expression was found in the brainstem and spinal cord. Several bands were found in Western blots which were all absent/markedly attenuated in blots incubated with antigen-absorbed antibody, indicating specificity of the antibody: two bands of 38 and 39 kDa represent the non-glycosylated form of FPR2. The 38 kDa band is consistent with the expected molecular weight of FPR2, and the diffuse 39 kDa band is also likely a non-glycosylated form of FPR2, while the 55 kDa band is likely a glycosylated form of the receptor. FPR2 is known to be glycosylated [46] and conserved *N*-glycosylation sites are found on the human FPR2 protein at the N-terminus (Asn4) and second extracellular loop (Asn179) [47]. It is likely the rat ortholog possesses these sites as well. A wide range of molecular weights of FPR2 on SDS–PAGE gels has been reported in the literature, ranging from 38 to 100 kDa, which is attributed to glycosylation or dimerization [25, 48, 49]. More than a single band of protein is also found in Western blots of FPR1, which is attributed to a mixture of non-glycosylated and differently-glycosylated forms of the receptor [50, 51]. Glycosylation is necessary for the proper folding of FPR1 [51], and this is likely also for FPR2.

Immunohistochemical analyses showed absence of staining in sections that had been incubated with antigen-absorbed antibody, indicating specificity of immunolabelling. Consistent with the RT-PCR results, generally denser staining was observed in the brainstem and spinal cord than the forebrain. The cerebral cortex was moderately densely immunolabelled for FPR2, and staining was observed as punctuate profiles in the neuropil. The hippocampus was lightly stained, except for dentate granule neurons and their axons (mossy fibres), which were densely labelled. These findings are congruent with studies that show that FPR2 is expressed in hippocampal neural stem cells [44, 45]. Since the dentate gyrus -> CA3 projection is part of a trisynaptic circuit through the hippocampus for consolidation of long term memory [52], this suggests an important role of FPR2 in memory formation. The thalamus was lightly labelled, while the hypothalamus was moderately densely labelled. The latter might account for the high levels of FPR2 mRNA and protein detected in this region by RT-PCR and Western blots. The deep cerebellar nuclei, the inferior olivary nucleus and the vestibular nuclei in the brainstem were densely labelled for FPR2. This suggests that FPR2 could be important in balance and motor coordination. The high level of FPR2 immunoreactivity in the deep cerebellar nuclei, and FPR2 protein in western blots of the cerebellum, could be due to a high level of translation, in view of the low level of mRNA expression in the cerebellum. FRP2 immunolabel was also observed in the spinal trigeminal nucleus and the dorsal horn of the spinal cord, which are sites of termination of primary nociceptive afferents. This suggests a role of FPR2 in the ascending pain pathway. LXA4 has been shown to improve learning and memory after subarachnoid haemorrhage, and these effects were mediated by FPR2 [22]. In addition, protective effects of RvD1 against surgery-induced memory loss have been reported [53]. The findings in this study on the rat CNS are consistent with results of a broad survey of FPR2/FPR-L1 immunolocalization at the light microscopic level in human organs. In that study, dense staining was reported (but not illustrated) in the cerebellar system, including the inferior olivary nucleus, vestibular nuclei, spinal trigeminal nucleus and dorsal horn of the spinal cord [32], reviewed in [54]. Previous studies have shown that Aβ oligomers accelerates senescence in adult hippocampal neural stem/progenitor cells via FPR2, and effect of LXA4 in reducing Aβ toxicity in the mouse brain [55, 56].

In the present study, electron microscopy showed FPR2 immunolabel in axon pre-terminals that did not form synapses with dendrites, but could instead represent growth processes. This was tested by examining the effect of FPR2 inhibition in primary cultured neurons. Incubation of cells with concentrations of FPR2 inhibitors PBP10 or WRW4, that did not cause loss in cell viability, showed that inhibition of FPR2 signalling significantly diminished the lengths of axons (labelled by Tau antibody) and dendrites (labelled by MAP2 antibody), indicating that FPR2 is involved in axonal and dendritic outgrowth. Changes in neuronal polarity were also found (data not shown). These results are consistent with findings that resolvin D1 stimulates neurite outgrowth of primary cultured dorsal root ganglion cells from normal mice [57]. They are also in congruence with studies which show that FPR2 promotes neuronal differentiation, with more and longer primary neurites per cell [44, 45].

The findings of dense FPR2 immunolabelling in the brainstem and cerebellum are consistent with our previous findings that that the enzyme that catalyses the release of arachidonic acid from membrane phospholipids, cytosolic phospholipase A_2_ (cPLA_2_) is also present at high levels in the hindbrain [58] and spinal cord [59]. Arachidonic acid can be further metabolized by lipoxygenases Alox5 and Alox12 to form LXA4 [60]. It is striking that both cPLA_2_ and FPR2 are present at high levels in almost the same nuclei in the hindbrain. For example, the deep cerebellar nuclei, dorsal and ventral cochlear nuclei, spinal trigeminal nucleus, and dorsal horn of the spinal cord contain high levels of immunostaining to both cPLA_2_ [58, 59] and FPR2 (this study). These observations suggest that the major ligand of FPR2 in the normal brainstem and cerebellum is LXA4.

In contrast to the brainstem and spinal cord, the cerebral cortex and hippocampus contain much lower levels of cPLA_2_ expression [58]. However, another PLA_2_ isoform that is present at high levels in the cortex, which could also release arachidonic acid for LXA4 formation, is secretory phospholipase A_2_ XIIA (sPLA_2_ XIIA) [61]. Lipidomic analyses of the prefrontal cortex after antisense knockdown of sPLA_2_ XIIA reveal changes in phospholipid and lysophospholipid species, which are consistent with a role of this enzyme in the endogenous release of arachidonic acid. Antisense oligonucleotide knockdown of sPLA_2_ XIIA in the prefrontal cortex results in deficits in the attention set shifting task [61].

RvD1 is also a ligand for FPR2. RvD1 interacts with FPR2 [23], and attenuates inflammation and promotes functional recovery after focal brain damage [62]. The enzyme calcium independent phospholipase A_2_ (iPLA_2_) preferentially releases DHA from brain membrane phospholipids. DHA is metabolized by Alox15 and Alox5 to RvD1. Both iPLA_2_ [63] and Alox15 [33] are expressed at high levels in the forebrain, including the cerebral cortex. Hence, it is possible that besides LXA4, RvD1 is a ligand for FPR2 in the cerebral cortex.

In conclusion, the present study adds to recent findings that show an important role of anti-inflammatory molecules in neural signalling [33], by demonstrating that a receptor for lipoxin A4 and resolvin D1, FPR2, is present in specific locations in the brain and spinal cord. Further studies are necessary to elucidate the in vivo function of FPR2 in the CNS.
